# Monitoring What Governments “Give for” and “Spend on” Vaccine Procurement: Vaccine Procurement Assistance and Vaccine Procurement Baseline

**DOI:** 10.1371/journal.pone.0089593

**Published:** 2014-02-20

**Authors:** E. A. S. Nelson, David E. Bloom, Richard T. Mahoney

**Affiliations:** 1 Department of Paediatrics, Faculty of Medicine, The Chinese University of Hong Kong, Hong Kong SAR, People's Republic of China; 2 Department of Global Health and Population, Harvard School of Public Health, Boston, Massachusetts, United States of America; 3 Global Health Consultant, Sedona, Arizona, United States of America; Thomas Jefferson University, United States of America

## Abstract

**Background:**

The Global Vaccine Action Plan will require, *inter alia,* the mobilization of financial resources from donors and national governments – both rich and poor. Vaccine Procurement Assistance (VPA) and Vaccine Procurement Baseline (VPB) are two metrics that could measure government performance and track resources in this arena. VPA is proposed as a new subcategory of Official Development Assistance (ODA) given for the procurement of vaccines and VPB is a previously suggested measure of the share of Gross Domestic Product (GDP) that governments spend on their own vaccine procurement.

**Objective:**

To determine realistic targets for VPA and VPB.

**Methods:**

Organization for Economic Co-Operation and Development (OECD) and World Bank data for 2009 were analyzed to determine the proportions of bilateral ODA from the 23 Development Assistance Committee (DAC) countries disbursed (as % of GDP in current US$) for infectious disease control. DAC country contributions to the GAVI Alliance for 2009 were assessed as a measure of multilateral donor support for vaccines and immunization programs.

**Findings:**

In 2009, total DAC bilateral ODA was 0.16% of global GDP and 0.25% of DAC GDP. As a percentage of GDP, Norway (0.013%) and United Kingdom (0.0085%) disbursed the greatest proportion of bilateral ODA for infectious disease control, and Norway (0.024%) and Canada (0.008%) made the greatest contributions to the GAVI Alliance. In 2009 0.02% of DAC GDP was US$7.61 billion and 0.02% of the GDP of the poorest 117 countries was US$2.88 billion.

**Conclusions:**

Adopting 0.02% GDP as minimum targets for both VPA and VPB is based on realistic estimates of what both developed and developing countries should spend, and can afford to spend, to jointly ensure procurement of vaccines recommended by national and global bodies. New OECD purpose codes are needed to specifically track ODA disbursed for a) vaccine procurement; and b) immunization programs.

## Introduction

In December 2010, global health leaders committed to making the next 10 years the Decade of Vaccines – to ensure discovery, development, and delivery of lifesaving vaccines globally, especially for the benefit of the poorest countries [1]. To meet the goals of the Decade of Vaccines (2011–2020), Ministers of Health from 194 countries endorsed a Global Vaccine Action Plan in May 2012 [2]. This plan calls for increased funding for immunization, including commitments by governments to invest in immunization commensurate with their ability to pay. It also calls for efforts to seek funds from new domestic sources as well as from international donors.

The Vaccine Procurement Baseline (VPB) has been previously suggested as a strategy to enhance transparency, equity, and sustainability in funding vaccine procurement for immunization programs [3]. Based on an analysis of Gross Domestic Product (GDP), population, and crude birth rate in countries with approximately $500 GDP per capita, it was shown that about 0.01% of GDP would be required to purchase all Expanded Program on Immunization vaccines in 1998. During the same period, developed countries allocated in excess of 0.01% of GDP (US, 0.035%; UK 0.0163%; Canada 0.0175%) to provide the traditional Expanded Program on Immunization vaccines plus a number of new vaccines. The original VPB proposal required that all countries spend a minimum of 0.01% of GDP on vaccine procurement, and that if recommended vaccines could not be obtained with those funds, the balance would be paid by external sources [3].

Official Development Assistance (ODA) is described as “*[f]lows of official financing administered with the promotion of the economic development and welfare of developing countries as the main objective, and which are concessional in character with a grant element of at least 25 percent (using a fixed 10 percent rate of discount). By convention, ODA flows comprise contributions of donor government agencies, at all levels, to developing countries (“bilateral ODA”) and to multilateral institutions. ODA receipts comprise disbursements by bilateral donors and multilateral institutions. Lending by export credit agencies – with the pure purpose of export promotion – is excluded*” [4]. In October 1970 the United Nations General Assembly passed resolution 2626, which included the goal that “*each economically-advanced country will progressively increase its official development assistance to the developing countries and will exert its best efforts to reach a minimum net amount of 0.7% of its gross national product at market prices by the middle of the Decade*” [5]. Sweden and the Netherlands were first to achieve this target in 1975, followed by Norway (1976) and Denmark (1978) [5].

Immunization is well accepted as one of the most cost-effective of all health interventions [2], [6]. However, it is likely that the economic benefits of investing in vaccination programs have been underestimated since traditional economic and disease reduction evaluations do not consider the following: healthy children perform better at school; healthy adults are more productive and better able to care for their children's health and education; healthier families are more likely to save for the future and to have fewer children; and healthier societies are more likely to attract more foreign direct investment including tourism [7], [8]. Failure to consider these broader benefits of vaccination could prevent the benefits of immunization being fully realized [9]. Recent estimates suggest that over the decade to 2020, immunization will save more than US$ 2.6 billion in averted treatment costs, lost caretaker wages and lost productivity in the world's poorest countries [6].

**Figure 1:**
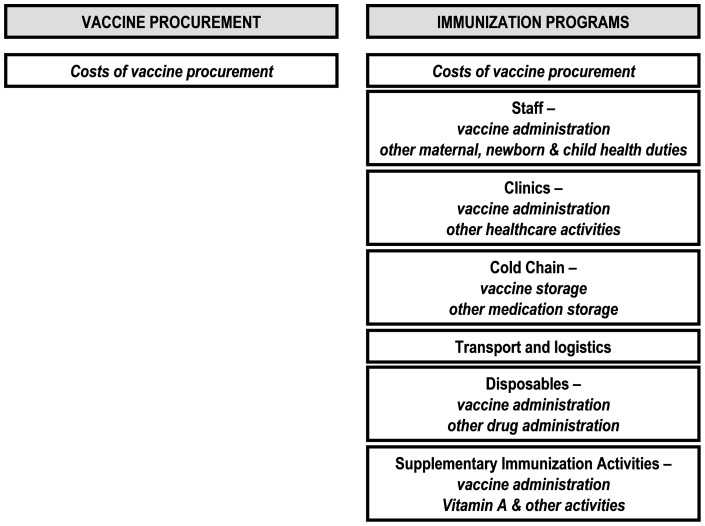
Monitoring costs to fully vaccinate a child requires monitoring the costs of both vaccine procurement and the costs of providing immunization programs.

Monitoring costs to fully vaccinate a child requires monitoring the costs of both vaccine procurement and the costs of providing immunization programs (Figure 1). This study focuses on the cost of vaccine procurement for routine immunization only. Delivery costs and costs (vaccine and delivery) for catch-up immunization, when necessary, are important, but policy makers are often first concerned with the per dose cost of a vaccine for routine immunization. This is because, for new vaccines, the per dose costs are often an order of magnitude greater than the estimated delivery costs and the routine immunization vaccine costs will most often extend indefinitely while catch-up can be completed in a few years. Also it is likely that monitoring expenditures for immunization programs will be significantly more challenging than monitoring expenditures on vaccine procurement, since immunization programs overlap with other maternal, newborn, and child health (MNCH) services (Figure 1).

We herein analyze data from the Organization for Economic Co-Operation and Development (OECD) and the World Bank to determine the total proportion of bilateral ODA disbursed in 2009 as % of 2009 GDP in current US$, the proportion allocated for social programs in the least developed countries (LDC), and the proportion of bilateral ODA allocated to infectious disease control (as an approximate estimate of bilateral ODA currently allocated for immunizations). Data from the GAVI Alliance (GAVI) are assessed to obtain a lower-bound estimate of current multilateral contributions made by donor countries for vaccines procurement and strengthening immunization programs. We propose to combine the previous concept of VPB (share of GDP allocated by national governments for vaccine procurement) with a new concept of Vaccine Procurement Assistance (VPA), where VPA would be defined as official financing, via grants, for vaccine procurement.

## Methods

### OECD data

The OECD website provides a detailed database of disbursements made by the 23 Development Assistance Committee (DAC) countries that report to the Creditor Reporting System [10]. Gross disbursements (in constant 2009 US$) for bilateral ODA were downloaded on 20 December 2011. The Creditor Reporting System database has selectable filters on the website that allowed the following donor country comparisons to be made:

1. Total bilateral ODA versus bilateral ODA grants (using filter “flow”);
2. Total bilateral ODA to all 172 pre-selected recipient countries versus bilateral ODA only to the LDCs (using filter “income group”);
3. Total bilateral ODA versus bilateral ODA for social infrastructure and services (using filter “sector”);
4. Total bilateral ODA versus bilateral ODA only to public sector (using filter “channel”);
5. Total bilateral ODA versus bilateral ODA only for infectious disease control (using filter “purpose code”  = 12250).

Infectious disease control includes ODA disbursed for (a) immunization; and (b) prevention and control of infectious and parasitic diseases, but excludes ODA for malaria control, tuberculosis control, and control of HIV/AIDS and other sexually transmitted diseases). Purpose code 12220 for basic health care includes ODA for: basic and primary health care programs; paramedical and nursing care programs; supply of drugs, medicines and vaccines related to basic health care. However only the infectious disease control filter was used to provide an approximate estimate of bilateral ODA that a country might have allocated for immunization programs, since the basic health care filter was considered too broad.

### GAVI data

Since 2000, GAVI has been the main multilateral agency that provides funding for vaccine procurement for the LDCs. DAC country contributions to GAVI for 2009 [11], thus serve as an approximate lower estimate of multilateral ODA allocated for vaccine procurement. These GAVI contributions included direct contributions, contributions to the International Finance Facility for Immunization, contributions to the Advance Market Commitment program, and contributions to GAVI's Matching Fund. Thus, although bilateral ODA for infectious disease control and GAVI contributions do not provide the full picture of donor country support for vaccine procurement, these data provide some perspective of what could be realistic contributions that countries could make as a proportion of their GDP.

### World Bank data

**Figure 2:**
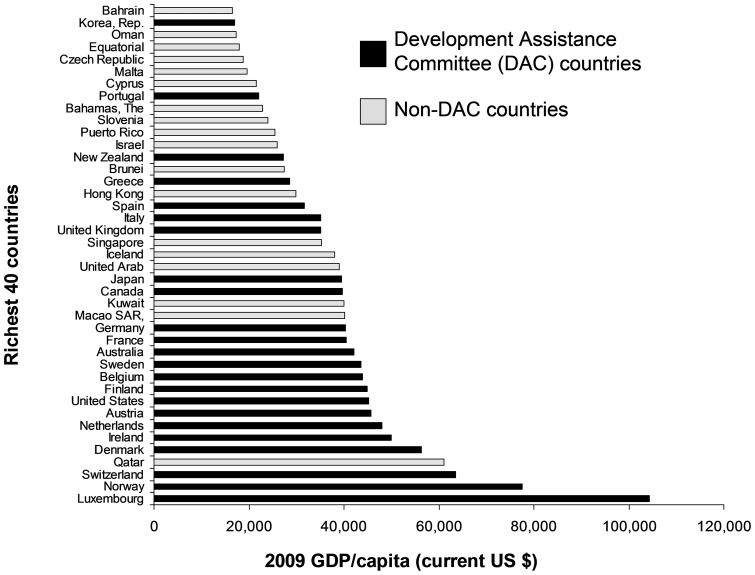
Richest 40 countries in 2009 based on gross domestic product (GDP) per capita measured in current US$.

World Bank data allow access to a number of different databases searchable according to various criteria [12]. Using the World Development Indicators and Global Development Finance database, the following country-level data for 2009 were downloaded for countries: total population; GDP (current US$); GDP, purchasing power parity (current international $). The richest 40 territories or countries were ranked according to their GDP per capita (current US$) (Figure 2). Affluent non-DAC countries were excluded from further analysis as ODA data on these countries are not included in the OECD Creditor Reporting System database. ODA and the various sub-categories of ODA were calculated for each country as % of GDP (current US$).

## Results

**Figure 3:**
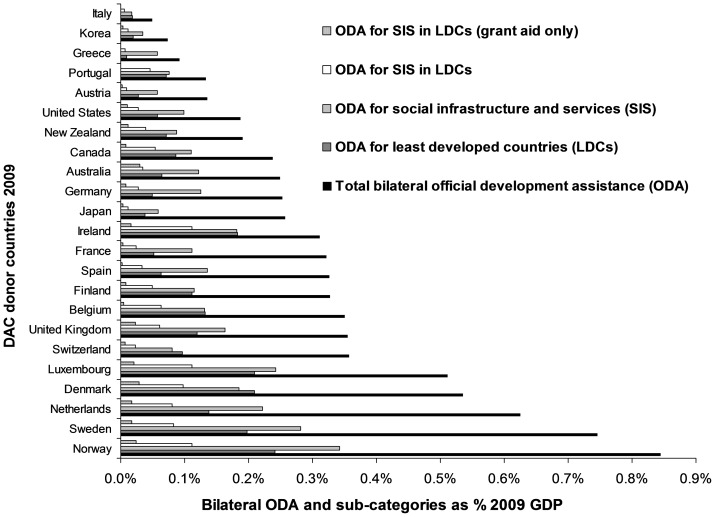
Sub-categories of bilateral official development assistance in 2009 for the 23 Development Assistance Committee (DAC) countries as a share of their gross domestic product (GDP) in current 2009 US$.

In 2009 only two countries (Norway and Sweden) disbursed more than 0.7% of their GDP as bilateral ODA (Figure 3) [5], and only three countries (Luxembourg, Norway and Ireland) gave more than 0.1% of their GDP for social programs in the LDCs as part of their bilateral ODA programs (Figure 3) [13]. However, if only the grant aid disbursed for social programs in the LDCs is considered, then no country achieved a level of even half of 0.1% of GDP for this form of ODA (Figure 3).

In 2009 global GDP was US$ 57.9 trillion [12], and DAC GDP was US$ 38.1 trillion [10]. In the same year, total DAC bilateral and multilateral ODA was US$ 134 billion (0.23% of global GDP and 0.35% of DAC GDP) and DAC bilateral ODA was US$ 94.7 billion (0.16% of global GDP and 0.25% of DAC GDP). DAC bilateral ODA was 70.7% of total DAC ODA for 2009.

**Figure 4:**
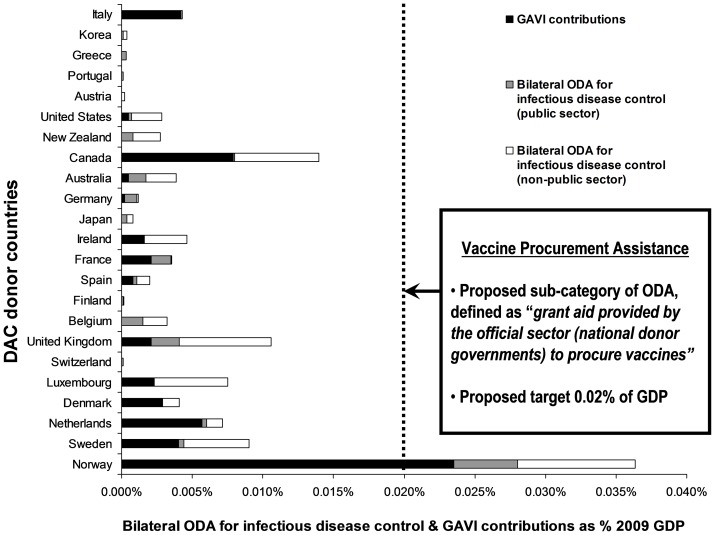
Bilateral official development assistance (ODA) allocated for infectious disease control and contributions to the GAVI Alliance (GAVI) in 2009 for the 23 Development Assistance Committee (DAC) countries as a share of their gross domestic product (GDP) in current 2009 US$. Countries ranked according to total bilateral ODA as a proportion of GDP disbursed in 2009.

The greatest proportion of their GDP for infectious disease control (OECD purpose code 12250) was given by Norway (0.013%), followed by the United Kingdom (0.0085%) (Figure 4). Although government (public sector) ownership of national immunization programs might be anticipated to provide the most equitable and comprehensive provision of services, less than half of the bilateral ODA disbursed for infectious disease control was allocated to the public sector (Figure 4). In 2009 GAVI received US$ 676.1 million in contributions; 82% of this came from 14 DAC countries, with the majority coming from seven: Canada (19.0%), Norway (15.9%), Italy (15.8%), United States of America (13.5%), France (10.1%), the Netherlands (8.2%), and United Kingdom (8.1%) [11]. As a proportion of their GDPs, Norway (0.024%), Canada (0.008%) and the Netherlands (0.006%) gave the greatest contributions to GAVI in 2009 (Figure 4). We combined contributions to GAVI and bilateral ODA for infectious disease control for 2009 to explore what could be a realistic target for VPA. The greatest proportion of their GDP for these combined contributions was given by Norway (0.036%), Canada (0.014%) and the United Kingdom (0.011%).

The WHO-UNICEF Global Immunization Vision and Strategy has a goal of reducing mortality due to vaccine-preventable diseases by two-thirds by 2015 [14]. This will require scaling up use of traditional and underused vaccines, as well as the introduction of new vaccines. The estimated cost of doing this for the 10-year period 2006 to 2015 was US$ 76 billion for 72 GAVI-eligible (for 2005–2010 included countries with 2003 gross national income per capita < US$ 1000) and 45 low- and lower-middle income countries, i.e., US$ 7.6 billion/year. Costs included in this study were baseline costs, vaccine costs, systems costs and campaign costs. If only current routine immunization is maintained then 25% of the costs were for vaccines, but with scaling up, 60% of the costs were for vaccines. On an annual basis for 2009 this US$ 7.6 billion/year equated to 0.107% of GDP for the 72 GAVI-eligible countries and 0.038% of GDP for the 45 low- and lower-middle-income countries (Table 1). To make an approximate estimate of the VPB costs for the upper-middle-income and DAC countries, we assumed that with tiered pricing the costs would be a similar proportion of GDP/capita (2.11%) and respectively equate to US$255 and US$843 per child, 0.045% and 0.03% as % of GDP, and 2.15 billion and 11.5 billion in total costs (Table 1). Although very approximate, these estimates are within previous ranges [3].

**Table 1:** Estimated costs of achieving WHO-UNICEF Global Immunization Vision and Strategy by scaling up use of traditional, underused and new vaccines in GAVI-eligible and low- and lower-middle income countries [14] and extrapolated costs for upper-middle-income and Development Assistance Committee (DAC) countries as percentage of 2009 gross domestic product (GDP) measured in current US$.

	Total cost of vaccines and immunization per year US$ billion	Cost of vaccines and immunization as % 2009 GDP	Cost to fully vaccinate a child US$	Cost to fully vaccinate a child as proportion of 2009 GDP per capita
Poorest 117 countries [14]	7.6 (2.3–11)	0.053% a	56	2.11% a
72 GAVI-eligible countries [14]	3.5 (1.3–4)	0.107% a	39	3.56% a
45 low- and lower-middle- income countries [14]	4.2 (1.1–7)	0.038% a	92	2.02% a
Upper-middle- income countries	2.15 b	0.045% b	255 b	2.11% b
DAC countries	11.5 b	0.03% b	843 b	2.11% b
Total	21.4 a ^,^ b	0.037% a ^,^ b		

Footnotes:

a Birth data missing for Dominica, Kiribati, Palau, Marshall Islands, Seychelles, St. Kitts and Nevis, and Tuvalu and GDP data missing for Cuba, Democratic People's Republic of Korea, Marshall Islands, Myanmar, Somalia and Tuvalu.

b Assumes that with tiered pricing the cost to vaccinate a child in upper-middle-income and DAC countries would also be 2.11% of GDP per capita.

## Discussion

We propose that VPA be a clearly defined and specific subcategory of ODA and that all donor governments commit to allocating more than 0.02% of GDP for vaccine procurement for recipient countries. A target of 0.02% is also proposed for VPB, i.e. the share of GDP spent on vaccine procurement by all national governments. Monitoring VPA and VPB, and reporting which rich countries give less than 0.02% of their GDP for vaccine procurement in poorer countries and which national governments spend less than 0.02% of GDP on their own vaccine procurement, could be a powerful advocacy tool to encourage greater giving and greater spending. Conceptually, the benchmark of 0.02% of GDP for both VPA and VPB provides a starting point for realistic funding targets. However with potential changes in the near future, such as improved documentation of expenditures for vaccine procurement and immunization programs, with the development of new vaccine technologies and delivery systems, and with increased experience with a variety of funding mechanisms, it is likely that the 0.02% targets for VPA and VPB will need to be adjusted upwards or downwards in the future. We used cost estimates for 2006 to 2015 for the 117 poorest countries [14], but more recent estimates for 2011 to 2020 for 94 low- and lower middle-income countries suggest that costs could be only US$ 5.75 billion per year during the Decade of Vaccines [15]. Bill Gates has suggested that “Setting clear goals and finding measures to mark progress, together with raising the funds for health and development projects will be necessary to sustain the momentum of the past 15 years in improving lives of the poorest” [16]. We believe that presenting costs of vaccine procurement and immunization programs in terms of a percentage of GDP provides simple and transparent measures to mark progress, as well as providing a tool to advocate for more funds.

Recent analyses have assessed ODA spending in relation to MNCH [17]–[20]. Although there have been increases to total worldwide ODA, including that allocated to MNCH, these absolute increases were not linked to increases in donor GDP [20]. In 2009 total bilateral and multilateral ODA in US$, excluding debt forgiveness, was US$ 132.36 billion (12.5% for health; 4.9% for MNCH; 3.7% for MNCH in the 74 priority countries with high child mortality as tracked by the “Countdown to 2015 Initiative”; and 0.48% (0.6382 billion) for immunization in these 74 countries) [20]. With DAC total ODA representing 0.35% of DAC GDP in 2009, we can estimate that in 2009 VPA for the 74 priority countries could have been no more than 0.0017% of DAC GDP (0.6382/[132.36/0.0035]). However, in 2009 Norway gave 0.024% of its GDP directly to GAVI, showing that a 0.02% VPA target is achievable for countries that are committed to it (Figure 4). This contribution was also possible at the height of the Global Financial Crisis when it is likely that some countries were reducing their ODA. For this reason it is also possible that the 2009 figures used in our analysis may illustrate an under-, rather than overestimate of typical annual ODA.

Donor funds for procurement of children's vaccines are needed for three reasons. First, they address the shared global responsibility for achieving equity between developed and developing countries. Children in industrialized countries receive new vaccines as soon as they become available, whereas this is not the case in many developing countries. A system-wide change is required to achieve equity. Second, vaccines are an international public commodity (a public good). Responsibility for health may lie at the national government level but the determinants of health and the means for governments to fulfill that responsibility are increasingly global. The third reason has to do with the economics of the vaccine industry. For vaccines, the greater the volume or number of manufacturers, the lower will be the price. Industry cites the high cost of product development and capital investment for production facilities as rationales for high vaccine prices, but extremely high profit margins in the pharmaceutical industry and high expenses on product promotion raise questions about these rationales.

A 2001 survey reported that 70% to 90% of the public from 13 DAC countries supported the principle of providing ODA to developing countries [21]. Yet there appear to be major misconceptions about the amount of ODA allocated, with a recent US survey showing that the American public vastly overestimated the amount of US foreign aid – believing it in the region of 25% of the federal budget when in fact it was only 1% [22]. The respondents considered that about 10% of the federal budget would be a reasonable allocation for aid. We can speculate how the public would react to these questions if they realized that most ODA is not allocated for poverty alleviation, with only a relatively small fraction of ODA going to social programs in the poorest countries (Figure 3). Although most donors in 2009 gave all or the majority of the ODA as grant aid (data not shown), there were still some countries giving some ODA as repayable loans. One might anticipate that there could be strong public support for donor governments to disburse 0.1% of GDP for genuine poverty alleviation programs [13], and many people, if genuinely informed, might wish to see the full amount of recommended ODA (0.7% of GDP [5]) targeted predominantly for social programs in the LDCs. More needs to be done to clearly inform the public in these countries that the proportion of their ODA going to genuine poverty alleviation is only a small fraction of total ODA.

VPB was proposed as a basic parameter to guide the allocation of donor funds in an equitable and transparent way [3]. We propose revising the VPB target upwards from 0.01% to 0.02% of GDP to reflect a more realistic goal of the costs of including new vaccines in national immunization programs (Table 1). Monitoring VPB and reporting those national governments (both rich and poor) that do not provide all recommended vaccines in their national immunization programs, and yet spend less than 0.02% GDP for their own vaccine procurement (VPB) could be another important advocacy tool for non-government organizations, patient groups, and parents. To accurately monitor VPB, it will be necessary for all governments to report the amount spent on their own vaccine procurement for their publicly funded immunization programs. WHO reports that it will track and monitor resources invested in immunization on a yearly basis during the Decade of Vaccines using the newly revised framework of the OECD/EUROSTAT/WHO System of Health Accounts [23], [24]. Within this framework expenditures on immunization programs will be monitored – both the time and skills of personnel, as well as the purchase of vaccines (Figure 1).

We argue that, while it is important to monitor immunization program costs, a focus on vaccine procurement is justified because the vaccines are the essential means to provide protection. A country can have a richly endowed immunization system, but this is meaningful only when there are vaccines to deliver. Closely tracking vaccine procurement costs could also provide a monitoring and advocacy tool to drive down vaccine prices. However, because adequate operational costs are also essential to protect the investment in vaccines and improve program performance [25], it would be desirable at a later point to expand the concept of VPA and VPB and define two new, broader categories:

1. Immunization Program Assistance: official grant financing from donor governments given for immunization programs in developing countries.
2. Immunization Program Baseline: share of GDP that governments spend on their own immunization programs.

**Figure 5:**
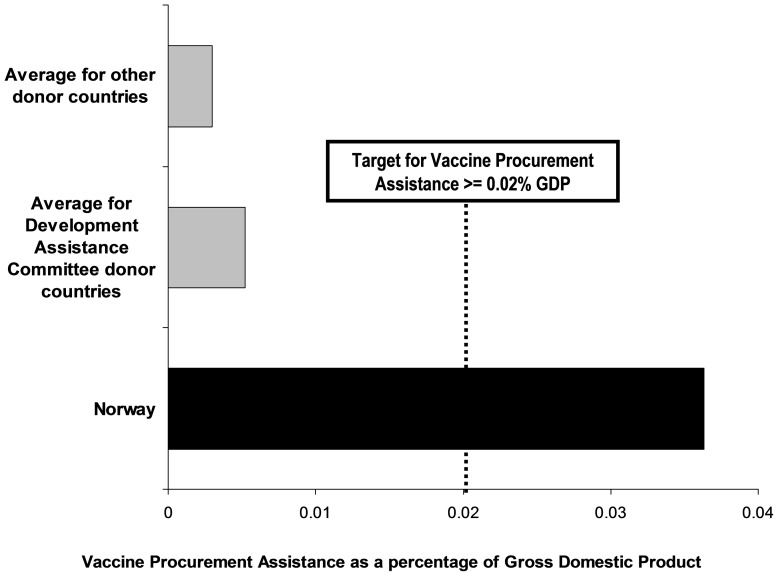
Example of a score card to show how Norway is performing relative to other countries for its Vaccine Procurement Assistance, defined as “*grant aid provided by national donor governments to procure vaccines as a share of gross domestic product (GDP)*”.

**Figure 6:**
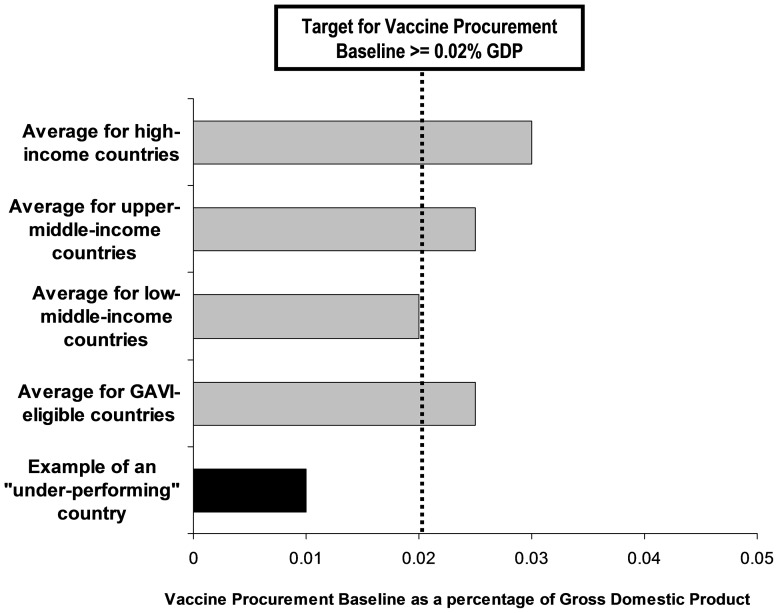
Example of a score card to show how a country of any income status is “under-performing” relative to others for the metric Vaccine Procurement Baseline, defined as “*share of gross domestic product spent on vaccine procurement*”.

The combined concepts of VPA and VPB could provide transparent metrics to show how much donor countries “give for” vaccine procurement (Figure 5) and how much all countries “spend on” vaccine procurement (Figure 6). The 0.02% of GDP minimum targets for both VPA and VPB are based on realistic estimates of what both developed and developing countries can likely afford. However, the proposed target for VPA should be seen as preliminary since an important limitation of our study is the absence of precise OECD purpose codes. Our estimates included funding specified for immunization programs (OECD purpose code 12250 for infectious disease control and GAVI contributions) but may have omitted some funding for vaccine procurement (OECD purpose code 12220 for basic health care). Since the current OCED purpose codes are too imprecise to accurately monitor VPA, it will be necessary for the OECD to provide new purpose codes that specifically document ODA disbursed for a) vaccine procurement and b) immunization programs. Although the complexities of monitoring diverse ODA expenditures should not be underestimated, particularly in view of the fact that they will vary between countries and over time, enhanced and more precise accounting of ODA should be achievable and will have the added benefit of providing greater transparency.

In 2009 0.02% of DAC GDP was US$ 7.61 billion (proposed VPA target) and 0.02% of the GDP of the poorest 117 countries was US$ 2.88 billion (proposed revised VPB target), which combines to US$ 10.5 billion and exceeds the US$ 7.6 billion required annually to achieve the Global Immunization Vision and Strategy for these 117 countries (Table 1). Documenting the share of GDP “given for” and “spent on” vaccine procurement could provide simple and transparent measures to monitor progress in the mobilization of the financial resources required to achieve the vision of the Decade of Vaccines and contribute to the Post-2015 Development Agenda.
